# Distilled Water

**Published:** 1888-11

**Authors:** 


### Distilled Water.

In his valuable "Manual," Adolph Vomacka says: It is not every water that is fit for use in the preparation of a pure distilled water, and certain it is that the so-called "distilled water," which is merely a by-product of other processes (condensation water from steam engines, for instance), is not fit to use in most of our pharmaceutical laboratory work. We must have for these, and especially for hypodermic injection solutions, an absolutely pure distilled water. To obtain this Vomacka recommends following the directions of the Pharmacopœia of the Netherlands, which are in effect as follows: Add to the water which you intend to distil potassium permanganate, so long as the violet color produced by each dosage is evanescent. When this is no longer the case, add a solution of alum until a weak acid reaction is obtained. Let the water stand until a precipitate no longer falls. Draw off the limpid portion and distill, throwing away the first and last thirds which pass over; in other words, distill one-third and throw away the distillate; distill, and reserve for use only one-half the remainder. This distillate may be relied on as chemically pure.—*National Druggist*, April, 1888.

